# Diagnostic value of medical thoracoscopy in malignant pleural effusion

**DOI:** 10.1186/s12890-017-0451-1

**Published:** 2017-08-04

**Authors:** Yan-Bing Wu, Li-Li Xu, Xiao-Juan Wang, Zhen Wang, Jun Zhang, Zhao-Hui Tong, Huan-Zhong Shi

**Affiliations:** Department of Respiratory and Critical Care Medicine, Beijing Institute of Respiratory Medicine and Beijing Chaoyang Hospital, Capital Medical University, Beijing, China

**Keywords:** Diagnosis, Malignant pleural effusion, Pleural biopsy, Sensitivity and specificity, Thoracoscopy

## Abstract

**Background:**

Medical thoracoscopy has been shown to be an efficacious procedure in diagnosing unexplained exudative pleural effusions with excellent safety. This study aimed to assess the diagnostic significance of thoracoscopy in the management of patients with malignant pleural effusion (MPE).

**Methods:**

Consecutive patients with malignant pleural effusion were retrospectively reviewed, and their demographic, radiographic, thoracoscopic and histological data were collected.

**Results:**

Between July 2005 and June 2014, 342 of 833 patients undergoing thoracoscopy were finally confirmed to suffer from MPE. The top three frequent causes of MPE were metastatic carcinoma (79.5%), malignant mesothelioma (10.2%), and lymphoma (2.9%). Among metastatic malignancies, the most common cancer was lung cancer (85.2%), followed by breast cancer (4.4%), ovarian cancer (2.2%), pancreatic cancer (1.8%), etc. No serious adverse events associated with thoracoscopy were recorded.

**Conclusions:**

Medical thoracoscopy is a valuable and safe tool in diagnosing malignant pleural effusion with minimal complication rates.

## Background

The identification of malignant cells in a pleural lavage in patients without pleural effusion suggests micrometastatic disease, and our previous meta-analysis [1] showed that positive pleural lavage cytological findings are associated with a higher recurrence rate and significant poorer survival, with the overall hazard ratio for patients having malignant cells in pleural lavage was 5.61 (95% confidence interval 3.98–7.90). In non-small-cell lung cancer patients, the evidence of even a minimal pleural effusion at diagnosis is an independent prognostic factor for worse survival [2]. Malignant pleural effusion (MPE) is frequently observed in multiple malignancies, and lung cancer is the most common cause [3]. The existence of MPE in patients indicates systemic dissemination of cancer and declining in life expectancy and quality [4, 5].

The current guideline recommended that thoracentesis and/or closed pleural biopsy can be used as the first diagnostic steps in the diagnosis of MPE [6]. However, these procedures usually do not work when pleural effusion with thickness less than 10 mm on chest computed tomography (CT) scans. Instead, the more invasive approaches, such as medical thoracoscopy (MT), can be considered to identify whether pleural biopsy contains malignant cells [3, 7]. As a matter of fact, MT is a highly sensitive and safe method for diagnosing exudative pleural effusions [8–10]. The recent developed semi-rigid MT is easy to use and can gain popularity among respiratory physicians who are accustomed to flexible bronchoscope [11, 12].

In the present retrospective study of patients with MPE having undergone at least one semi-rigid MT over a 9-year period in a Chinese 1600-bed general hospital, we analyzed the diagnostic efficiency and safety of MT in the diagnosis of MPE.

## Methods

The study protocol and ethical approval was approved by the Institutional Review Board for human studies of Beijing Chao-Yang Hospital, China. Informed consents were not required as this was considered a review of clinical practice.

Information including medical history, clinical presentation, laboratory examination results, and image data of unexplained exudative pleural effusions patients who underwent MT in our hospital between July 2005 and June 2014 were gathered, and only MPE patients were finally included in the current study. Unexplained exudative pleural effusions were defined as the patients underwent the initial diagnostic approaches including thoracentesis and/or closed pleural biopsy, and their diseases remain undiagnosed. The characteristics of the study population are listed in Table 1.

**Table 1:** Characteristics of the study population (*n* = 342)

Variables	Values
Age, yr., mean ± SD	62.8 ± 9.7
Sex, male/female, n (%)	183/159 (53.5/46.5)
Smoking status, n (%)	
Current or previous smoker	127 (37.1)
Non-smoker	149 (43.6)
Not clear	66 (19.3)
History of malignancy	32 (9.4)
CT imaging	
Mediastinal and hilum lymphadenopathy	196 (57.3)
Pleural thickening	159 (46.5)
Pulmonary consolidation or infiltration	158 (46.2)
Pulmonary mass or nodules	134 (39.2)
Pulmonary atelectasis	126 (36.8)
Pleural nodularity	51 (14.9)
Side of effusion, n (%)	
Right	149 (43.6)
Left	133 (38.9)
Bilateral	60 (17.5)
Size of effusion, n (%)	
Small	57 (16.7)
Moderate	44 (12.9)
Large	241 (70.4)
Effusion appearance, n (%)	
Blood- tinged	191 (55.9)
Yellow	151 (44.1)

MT procedures have been described in our previous publications [13, 14]. The diagnosis of MPE was established by the presence of the positive findings for malignancy in pleural biopsy.

Descriptive statistical methods were used in the data analysis (mean ± standard deviation [SD] or/and range).

## Results

Between July 2005 and June 2014, 833 patients with undiagnosed pleural effusions successfully underwent medical thoracoscopy [15]. Eventually, 342 patients with lymphocytic exudates were finally diagnosed with MPE; the mean age was 62.8 ± 9.7 years.

For 149 MPE patients, pleural fluid occurred only in the right side, for 133 only in the left, and for the rest 60 both sides were involved (Table 1). The size of a pleural effusion was clarified as small, moderate, or large based on CT imaging according to the methods described by Moy and colleagues [16]. In both unilateral and bilateral effusion, the proportions of small, moderate, and large size of pleural effusions were 16.7, 12.9, and 70.4%, respectively. The appearance of pleural effusion was blood-stained in 55.9% of patients, and in 44.1% was yellow.

In addition to pleural effusion, CT imaging revealed mediastinal and hilum lymphadenopathy, pleural thickening, pulmonary consolidation or infiltration, pulmonary mass or nodules, pulmonary atelectasis, and pleural nodularity (Table 1).

In all patients studied, we observed one or more abnormalities on the surface of parietal or/and visceral pleura under medical thoracoscopy. As shown in Table 2, pleural nodules, hyperemia, pleural adhesion, pleural plaques, ulcer, and the other pleural pathological changes were observed.

**Table 2:** Procedural details (*n* = 342)

Procedural details	Value
Pleural fluid removed, mL	1306.7 ± 753.0
Parietal pleura biopsies, n	10 ± 2
Thoracoscopic findings, n (%)	
Pleural nodules	243 (71.1)
Hyperemia	159 (46.5)
Pleural adhesion	125 (36.6)
Pleural plaques	69 (20.2)
Ulcer	10 (2.9)
Other pleural pathological changes	97 (28.4)

The most common etiological causes of MPE were metastatic carcinomas (*n* = 272), pleural malignant mesothelioma (*n* = 35), lymphoma (*n* = 10). It should be mentioned that we could not identify the original malignancies in 25 patients with MPE (Table 3). Among metastatic malignancies that resulted in MPE, the most common cancer included lung cancer, followed by breast cancer, ovarian cancer, pancreatic cancer (Table 4).

**Table 3:** Diagnoses established by thoracoscopy in patients with MPE (*n* = 342)

Diagnoses	n (%)
Non-small cell lung cancer	221 (64.6)
Adenocarcinoma	215 (97.3)
Squamous cell carcinoma	6 (2.7)
Small cell lung cancer	11 (3.2)
Other metastatic carcinoma	40 (11.7)
Malignant mesothelioma	35 (10.2)
Epithelioid	
Sarcomatoid	
Biphasic	
Undifferentiated	
Lymphoma	10 (2.9)
Undetermined	25 (7.3)

**Table 4:** Types of metastatic cancers (*n* = 272)

Metastatic cancers	n (%)
Lung cancer	232 (85.2)
Breast cancer	12 (4.4)
Ovarian cancer	6 (2.2)
Pancreatic cancer	5 (1.8)
Hepatic carcinoma	3 (1.1)
Esophageal carcinoma	2 (0.7)
Renal carcinoma	2 (0.7)
Thymic carcinoma	2 (0.7)
Gastric carcinoma	1 (0.4)
Myeloma	1 (0.4)
Sinuethmoid cancer	1 (0.4)
Malignant hemangioendothelioma	1 (0.4)
Base tongue carcinoma	1 (0.4)
Malignant melanoma	1 (0.4)
Cervical cancer	1 (0.4)
Endometrial cancer	1 (0.4)

No serious adverse events were observed, and transient chest pain (43.9%) induced by the indwelling chest tube was the most frequent minor complication. Subcutaneous emphysema was found in 8.5% of patients who recovered after chest-tube drainage. Minor bleeding was recorded in 6.4% of patients. And 5.6% of patients appeared with transient self-limited fever (38 °C or more).

## Discussion

Because the prognosis for patients with MPE is poor, an efficacious procedure that can establish a definite diagnosis as early as possible with a minimum of risk and discomfort would be highly desirable. If a patient with undiagnosed pleural effusion is suspected as malignant, cytologic examination of pleural fluid is the first recommendation [6]. Although repeated thoracenteses can enhance the sensitivity of cytology, it is usually only 50 to 70% [17]. When cytology fails, closed percutaneous needle biopsy was traditionally performed blindly by an Abrams or Ramel needle [18–20]. Nevertheless, its role in diagnosing MPE has been challenged, as the positive diagnostic rate of closed pleural biopsy was only about 50% [21, 22]. More recently, the real time image-guided pleural biopsy has been shown to be a promising procedure for sampling the pleura, since it can increase the sensitivity for diagnosing MPE to about 80% [21, 23–25].

Numerous tumor markers have been intensively examined for improving the diagnosis of MPE, however, seeking for a highly accurate pleural fluid tumor marker that reliably diagnoses MPE has been in vain so far [26]. Using one tumor marker alone for diagnosing MPE is not recommend according to the recent evidences, however, when combined two or more tumor markers together, the diagnostic sensitivity seems to be improved [27, 28]. The diagnostic performance of tumor markers for MPE seems to be similar with conventional tests including cytological examination–high specificity and low sensitivity. Tumor markers are less important in practice, since they do not complement the properties of conventional tests.

CT- or ultrasound-guided pleural biopsies are quite sensitive and safe, with the only reported complications being local hematoma and minor hemoptysis [21, 29]. The limitation of the image-guided pleural biopsy is the blindness of the procedure. MT overcomes this problem by allowing for the visualization of abnormal areas and for a direct biopsy, and thus improves the diagnostic accuracy of pleural effusions [8, 9]. Since June 2005, our institution started using MT as a routine method for patients with undiagnosed exudative pleural effusion in cases when either clinical, radiologic, laboratory, or cytologic investigation was failed. During a period of 9 years, 833 patients with unexplained pleural effusions underwent MT successfully, and among them, 342 were eventually diagnosed with MPE [15].

One or more abnormalities on the surface of parietal or/and visceral pleura were observed in the whole population in this study, including pleural nodules, hyperemia, pleural adhesion, pleural plaques, ulcer, and the other pleural pathological changes. Pathological examination revealed the presence of the positive findings for malignancy in pleural biopsy in 342 patients. The data in detail presented in the current study derived from our whole MT study population [15]. As reported in the previous publication [15], after a complete work-up including MT biopsies, the definite diagnoses of 92.6% (771/833) of patients with pleural effusions can be established definitely by MT followed by histopathological study.

It was noted that no etiological causes of pleural effusions can be identified in 7.4% (62/833) of patients even after MT [15]. All of these patients were followed up for at least 12 months, and did not complain about a new pleural effusion. No diagnosis other than benign pleural effusion was found in these 62 patients. Several studies suggest that among the patients with histological diagnosis of non-specific pleurisy made after MT, 8.3–18% of them were eventually diagnosed with MPE, usually pleural mesothelioma, during long term follow-up [30–32]. Therefore, we cannot exclude the possibility that a few patients with MPE-negative MT results would be finally diagnosed with MPE if we prolong the follow-up.

It has been reported that carcinoma from any organ can metastasize to the pleura, but lung, and breast carcinomas and lymphomas are the most common causes, digestive and ovary carcinomas are less frequent [33]. Our recent unpublished data indicated that during the past 3 years, 23.7% (365/1541) of pleural effusion patients admitted to our hospital were diagnosed with MPE. In the present study, our data showed that the most common etiological causes of MPE confirmed by MT were metastatic carcinomas, and followed by pleural malignant mesothelioma, lymphoma, and the other malignancies. Among metastatic malignancies that resulted in MPE, the most common cancer was lung cancer, followed by breast cancer, ovarian cancer, and pancreatic cancer.

MT has an excellent safety profile when performed by a trained physician, and a mortality rate associated with MT is always ≤0.8% [34]. A recent meta-analysis further suggested that mortality associated with MT was not observed, and that the major complication rate of MT was 1.5% and the minor complication rate was 10.5% [10]. In our 9-year study, MT procedures were well tolerated with a low rate of complications without serious adverse events, and transient chest pain caused by the indwelling chest tube was the most frequent minor complication. Large volumes of pleural fluid could be safely aspirated, although some patients suffered from coughing and chest discomfort after lung re-expansion with a chest tube.

The strengthen of this study was that study population was in a large size, in which 342 MPE patients were included. At the same time, our study also had limitations. First, as a retrospective study, it’s impossible to collect and analyze the required data in a prospective way. Second, we could only retrospectively reviewed the data from patients with MPE, and no data from the other control group, such as the patients with tuberculous pleurisy, were available, it was therefore not possible to calculate the sensitivity and specificity of MT in diagnosing MPE. Third, blind needle biopsies or image-assisted biopsies were performed only in a few patients before undergoing MT in our series. This partially explained why there were so many pleural effusion patients (833 cases) receiving MT examination in our institution during 9 years.

## Conclusions

In summary, MT is simple and safe with a high positive rate in the diagnosis of MPE. Due to its convenience and compatibility with existing bronchoscopy, MT appears to be a more widely performed procedure. Thus MT should be performed actively for proper patients with suspected MPE.

## Abbreviations

CT: Computed tomography
MPE: Malignant pleural effusion
MT: Medical thoracoscopy
SD: Standard deviation

## References

[1] Li YN, Shi HZ, Liang QL, Yang HB, Huang GM (2008). Prognostic significance of pleural lavage cytology in patients with lung cancer: a meta-analysis. Lung Cancer.

[2] Ryu JS, Ryu HJ, Lee SN, Memon A, Lee SK, Nam HS, Kim HJ, Lee KH, Cho JH, Hwang SS (2014). Prognostic impact of minimal pleural effusion in non-small-cell lung cancer. J Clin Oncol.

[3] Light RW (2002). Clinical practice. Pleural effusion. N Engl J Med.

[4] Sugiura S, Ando Y, Minami H, Ando M, Sakai S, Shimokata K (1997). Prognostic value of pleural effusion in patients with non-small cell lung cancer. Clin Cancer Res.

[5] Goldstraw P, Crowley J, Chansky K, Giroux DJ, Groome PA, Rami-Porta R, Postmus PE, Rusch V, Sobin L (2007). The IASLC lung cancer staging project: proposals for the revision of the TNM stage groupings in the forthcoming (seventh) edition of the TNM classification of malignant tumours. J Thorac Oncol.

[6] Roberts ME, Neville E, Berrisford RG, Antunes G, Ali NJ. Management of a malignant pleural effusion: British Thoracic Society pleural disease guideline 2010. Thorax. 2010;65(Suppl 2):ii32–40. 10.1136/thx.2010.136994 20696691

[7] Heffner JE (2008). Diagnosis and management of malignant pleural effusions. Respirology.

[8] Loddenkemper R (1998). Thoracoscopy--state of the art. Eur Respir J.

[9] Rodriguez-Panadero F (2008). Medical thoracoscopy. Respiration.

[10] Agarwal R, Aggarwal AN, Gupta D (2013). Diagnostic accuracy and safety of semirigid thoracoscopy in exudative pleural effusions: a meta-analysis. Chest.

[11] Ernst A, Hersh CP, Herth F, Thurer R, LoCicero J, Beamis J, Mathur P (2002). A novel instrument for the evaluation of the pleural space: an experience in 34 patients. Chest.

[12] Munavvar M, Khan MA, Edwards J, Waqaruddin Z, Mills J (2007). The autoclavable semirigid thoracoscope: the way forward in pleural disease?. Eur Respir J.

[13] Wang Z, Tong ZH, Li HJ, Zhao TT, Li XY, Xu LL, Luo J, Jin ML, Li RS, Wang C (2008). Semi-rigid thoracoscopy for undiagnosed exudative pleural effusions: a comparative study. Chin Med J.

[14] Wang Z, Xu LL, Wu YB, Wang XJ, Yang Y, Zhang J, Tong ZH, Shi HZ (2015). Diagnostic value and safety of medical thoracoscopy in tuberculous pleural effusion. Respir Med.

[15] Wang XJ, Yang Y, Wang Z, Xu LL, Wu YB, Zhang J, Tong ZH, Shi HZ (2015). Efficacy and safety of diagnostic thoracoscopy in undiagnosed pleural effusions. Respiration.

[16] Moy MP, Levsky JM, Berko NS, Godelman A, Jain VR, Haramati LB (2013). A new, simple method for estimating pleural effusion size on CT scans. Chest.

[17] Bennett R, Maskell N (2005). Management of malignant pleural effusions. Curr Opin Pulm Med.

[18] Abrams LD (1958). A pleural-biopsy punch. Lancet.

[19] Prakash UB, Reiman HM (1985). Comparison of needle biopsy with cytologic analysis for the evaluation of pleural effusion: analysis of 414 cases. Mayo Clin Proc.

[20] Renshaw AA, Dean BR, Antman KH, Sugarbaker DJ, Cibas ES (1997). The role of cytologic evaluation of pleural fluid in the diagnosis of malignant mesothelioma. Chest.

[21] Maskell NA, Gleeson FV, Davies RJ (2003). Standard pleural biopsy versus CT-guided cutting-needle biopsy for diagnosis of malignant disease in pleural effusions: a randomised controlled trial. Lancet.

[22] Chakrabarti B, Ryland I, Sheard J, Warburton CJ, Earis JE (2006). The role of Abrams percutaneous pleural biopsy in the investigation of exudative pleural effusions. Chest.

[23] Adams RF, Gray W, Davies RJ, Gleeson FV (2001). Percutaneous image-guided cutting needle biopsy of the pleura in the diagnosis of malignant mesothelioma. Chest.

[24] Benamore RE, Scott K, Richards CJ, Entwisle JJ (2006). Image-guided pleural biopsy: diagnostic yield and complications. Clin Radiol.

[25] Metintas M, Ak G, Dundar E, Yildirim H, Ozkan R, Kurt E, Erginel S, Alatas F, Metintas S (2010). Medical thoracoscopy vs CT scan-guided Abrams pleural needle biopsy for diagnosis of patients with pleural effusions: a randomized, controlled trial. Chest.

[26] Light RW (2004). Tumor markers in undiagnosed pleural effusions. Chest.

[27] Shi HZ, Liang QL, Jiang J, Qin XJ, Yang HB (2008). Diagnostic value of carcinoembryonic antigen in malignant pleural effusion: a meta-analysis. Respirology (Carlton, Vic).

[28] Liang QL, Shi HZ, Qin XJ, Liang XD, Jiang J, Yang HB (2008). Diagnostic accuracy of tumour markers for malignant pleural effusion: a meta-analysis. Thorax.

[29] Koegelenberg CF, Bolliger CT, Theron J, Walzl G, Wright CA, Louw M, Diacon AH (2010). Direct comparison of the diagnostic yield of ultrasound-assisted Abrams and Tru-cut needle biopsies for pleural tuberculosis. Thorax.

[30] Venekamp LN, Velkeniers B, Noppen M (2005). Does ‘idiopathic pleuritis’ exist? Natural history of non-specific pleuritis diagnosed after thoracoscopy. Respiration.

[31] Davies HE, Nicholson JE, Rahman NM, Wilkinson EM, Davies RJ, Lee YC (2010). Outcome of patients with nonspecific pleuritis/fibrosis on thoracoscopic pleural biopsies. Eur J Cardiothorac Surg.

[32] Metintas M, Ak G, Cadirci O, Yildirim H, Dundar E, Metintas S (2012). Outcome of patients diagnosed with fibrinous pleuritis after medical thoracoscopy. Respir Med.

[33] Marel M, Stastny B, Melinova L, Svandova E, Light RW (1995). Diagnosis of pleural effusions. Experience with clinical studies, 1986 to 1990. Chest.

[34] Michaud G, Berkowitz DM, Ernst A (2010). Pleuroscopy for diagnosis and therapy for pleural effusions. Chest.

